# Neuron-specific ablation of eIF5A or deoxyhypusine synthase leads to impairments in growth, viability, neurodevelopment, and cognitive functions in mice

**DOI:** 10.1016/j.jbc.2021.101333

**Published:** 2021-10-22

**Authors:** Rajesh Kumar Kar, Ashleigh S. Hanner, Matthew F. Starost, Danielle Springer, Teresa L. Mastracci, Raghavendra G. Mirmira, Myung Hee Park

**Affiliations:** 1 Molecular and Cellular Biochemistry Section, NIDCR, National Institutes of Health, Bethesda, Maryland, USA; 2 Division of Veterinary Resources, Diagnostic and Research Services Branch, National Institutes of Health, Bethesda, Maryland, USA; 3 NHLBI Murine Phenotyping Core, National Heart Lung and Blood Institute, National Institutes of Health, Bethesda, Maryland, USA; 4 Department of Biology, Indiana University-Purdue University-Indianapolis, Indianapolis, Indiana, USA; 5 Department of Medicine, The University of Chicago, Chicago, Illinois, USA

**Keywords:** eIF5A, deoxyhypusine synthase, post-translational modification, hypusine, translation, neurodevelopment, mouse genetics, neurodevelopmental disorder, cognitive function, CKO, conditional KO, DHPS, deoxyhypusine synthase, DOHH, deoxyhypusine hydroxylase, eIF5A, eukaryotic initiation factor 5A, MWM, Morris water maze, NW, North West

## Abstract

Eukaryotic initiation factor 5A (eIF5A)†, ‡ is an essential protein that requires a unique amino acid, hypusine, for its activity. Hypusine is formed exclusively in eIF5A post-translationally *via* two enzymes, deoxyhypusine synthase (DHPS) and deoxyhypusine hydroxylase. Each of the genes encoding these proteins, *Eif5a*, *Dhps*, and *Dohh*, is required for mouse embryonic development. Variants in *EIF5A* or *DHPS* were recently identified as the genetic basis underlying certain rare neurodevelopmental disorders in humans. To investigate the roles of eIF5A and DHPS in brain development, we generated four conditional KO mouse strains using the *Emx1*-*Cre* or *Camk2a*-*Cre* strains and examined the effects of temporal- and region-specific deletion of *Eif5a* or *Dhps*. The conditional deletion of *Dhps* or *Eif5a* by *Emx1* promotor–driven Cre expression (E9.5, in the cortex and hippocampus) led to gross defects in forebrain development, reduced growth, and premature death. On the other hand, the conditional deletion of *Dhps* or *Eif5a* by *Camk2a* promoter–driven Cre expression (postnatal, mainly in the CA1 region of the hippocampus) did not lead to global developmental defects; rather, these KO animals exhibited severe impairment in spatial learning, contextual learning, and memory when subjected to the Morris water maze and a contextual learning test. In both models, the *Dhps*-KO mice displayed more severe impairment than their *Eif5a*-KO counterparts. The observed defects in the brain, global development, or cognitive functions most likely result from translation errors due to a deficiency in active, hypusinated eIF5A. Our study underscores the important roles of eIF5A and DHPS in neurodevelopment.

Eukaryotic initiation factor 5A (eIF5A) is the only cellular protein that is activated by a unique post-translational modification that forms an unusual amino acid, hypusine [N^Ɛ^-(4-amino-2-hydroxybutyl)lysine] (1). Hypusine is essential for the activity of this factor. It is formed in the eIF5A precursor by two consecutive enzymatic steps (Fig. 1) (2). The first enzyme, deoxyhypusine synthase (DHPS) (3), catalyzes the transfer of the aminobutyl moiety from the polyamine spermidine to one specific lysine residue of the eIF5A precursor to form an intermediate, deoxyhypusine [N^Ɛ^-(4-aminobutyl)lysine] residue, which is subsequently hydroxylated by deoxyhypusine hydroxylase (DOHH) (4) to complete the synthesis of hypusine (Fig. 1). Homozygous, whole-body deletion of any of these three genes, *Eif5a*, *Dhps*, or *Dohh*, in mice causes early embryonic lethality (5, 6), and postnatal conditional deletion of *Eif5a* or *Dhps* leads to inhibition of organ development in mice (7, 8).

**Figure 1:**
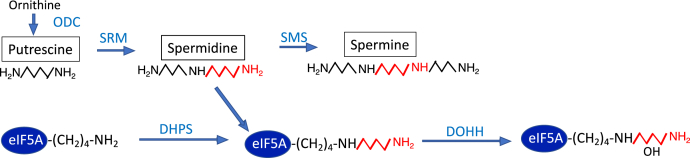
**The pathways of polyamine biosynthesis and hypusine modification in eIF5A.** The abbreviations are as follows: DHPS, deoxyhypusine synthase; DOHH, deoxyhypusine hydroxylase; ODC, ornithine decarboxylase; SMS, spermine synthase; SRM, spermidine synthase.

Polyamines (putrescine, spermidine, and spermine) are essential for eukaryotic cell growth and regulate a vast array of cellular activities (9, 10, 11). Polyamine homeostasis is tightly regulated by intricate mechanisms at multiple levels including biosynthesis, catabolism, and transport. The majority of cellular polyamines are bound to RNA, and the most important function of polyamines appears to be the regulation of translation as polycations (10, 12) and also as a component of hypusine in eIF5A. As hypusine is vital for eIF5A activity and cell proliferation, hypusine synthesis defines a critical function of polyamines in eukaryotic cell growth (13, 14).

In discrepancy to its nomenclature, eIF5A facilitates translation elongation rather than translation initiation (15, 16, 17). In yeast, eIF5A promotes translation elongation broadly at ribosome stall sites including sequences encoding polyproline stretches, and it also enhances translation termination (15, 18). eIF5A binds to the 80S ribosome between the peptidyl-tRNA site and the exit tRNA site (19). The hypusine side chain of eIF5A stabilizes the binding of the peptidyl tRNA to the 80S ribosome and facilitates peptide bond synthesis. Two or more eIF5A isoform genes have been identified in many eukaryotic organisms from fungi to humans. In the human and mouse, eIF5A2 shares 84% and 82% amino acid sequence identity with eIF5A1 (usually termed eIF5A), respectively. Both isoforms effectively undergo hypusine modification in cells (20).§ However, only eIF5A1 is constitutively expressed in all mammalian cells and tissues, whereas the eIF5A2 isoform mRNA expression appears to be tissue specific in the brain and testis (21). The eIF5A2 protein is normally undetectable in most mammalian tissues and cells, but increased expression of this isoform or eIF5A has been associated with various human cancers (22, 23, 24). The *Eif5a2* homozygous KO mouse develops and grows normally, suggesting that eIF5A2 is dispensable for mouse development (25).

DHPS is known to be totally specific for eIF5A (eIF5A1 and eIF5A2 isoforms); no other cellular protein is modified by DHPS. The exclusive specificity is based on the requirement for a macromolecular interaction between DHPS and the nearly intact N domain of eIF5A. A potential role of eIF5A and DHPS in neuronal growth and survival was first suggested in studies that used the neuronal cell line PC12 and rat primary hippocampal cultures *in vitro* (26). In these studies, a reduction of hypusinated eIF5A by using a DHPS inhibitor or DHPS RNAi attenuated neurite outgrowth and neuronal survival (26). Only recently, definitive genetic evidence for their importance in human neurodevelopment was reported (27, 28, 29). From whole-exome sequencing and genetic analysis, biallelic *DHPS* variants were identified as the cause of a rare autosomal recessive neurodevelopmental disorder (28). More recently, germ line, *de novo*, heterozygous *EIF5A* variants were also reported to be associated with a neurodevelopmental disorder (27). The patients carrying biallelic *DHPS* variants, or heterozygous *EIF5A* variants, share common phenotypes including intellectual disability and developmental delay. In addition, among the five *DHPS* variant patients, four have facial dysmorphism, one has microcephaly, and four have clinical seizures. Of the seven *EIF5A* variant patients, all display facial dysmorphism and five of them with microcephaly. Thus, a decrease in the biologically active, hypusinated form of eIF5A appears to interfere with proper neurodevelopment in humans.

To further investigate the roles of eIF5A and DHPS in brain development, we have generated four mouse strains in which either *Eif5a* or *Dhps* is deleted in the brain in a temporally and spatially specific manner using the *Emx1-Cre* or the *Camk2a-Cre* line. Phenotype analyses revealed severe morphological defects in the brain, growth retardation, and reduced viability in mice with *Emx1-Cre*–mediated deletion of *Eif5a* or *Dhps*, and impaired cognitive functions in mice with *Camk2a-Cre*–mediated deletion of *Eif5a* or *Dhps*.

## Results

### Generation of four conditional KO strains: *Eif5a*^*fl/fl*^;*Emx1-Cre* (*Eif5a*^*Emx*^), *Dhps*^*fl/fl*^;*Emx1-Cre* (*Dhps*^*Emx*^), *Eif5a*^*fl/fl*^;*Camk2a-Cre* (*Eif5a*^*Camk2a*^), and *Dhps*^*fl/fl*^;*Camk2a-Cre* (*Dhps*^*Camk2a*^)

**Figure 2:**
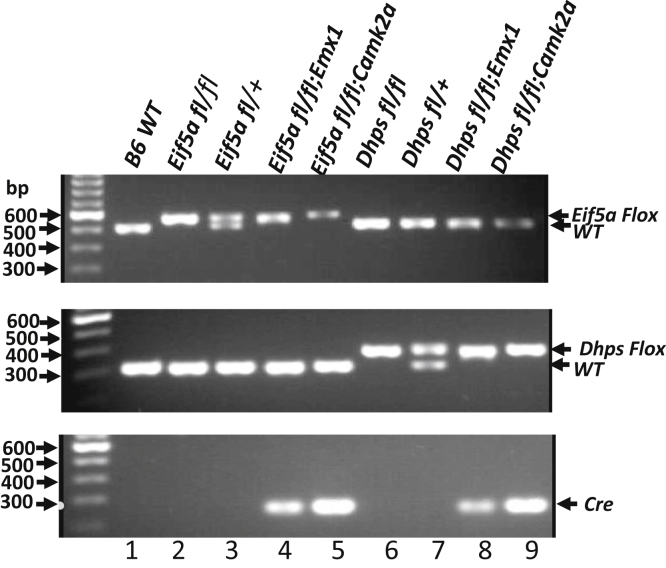
**Confirmation of genotypes of *Eif5a* or *Dhps* CKO mice by PCR.** The genomic DNA was isolated from ear punch tissues, and PCR was performed as described in Experimental procedures using the primer sets designed to identify the *Eif5a* floxed allele (*top panel*), the *Dhps* floxed allele (*middle panel*), and *Cre* transgene (*bottom panel*). The Thermo Fisher 100-bp DNA ladder is shown in the first lane of each gel. The PCR products of expected size (*Eif5a* Lox, 557 bp; *Eif5a* WT, 507 bp; *Dhps* Lox, 396 bp; *Dhps* WT, 319 bp; *Cre*, 300 bp, indicated by *arrows* on the *right side*) were detected for each strain. CKO, conditional KO.

*Emx1-Cre*–mediated KO of *Eif5a* or *Dhps* was achieved by two-step breeding. First, *Eif5a* ^*fl/fl*^ (8) or *Dhps* ^*fl/fl*^ (7) mouse was mated with *Emx1-IRES-Cre* mouse (30) to generate either *Eif5a* ^*fl/+*^*;Emx1-Cre* or *Dhps* ^*fl/+*^*;Emx1-Cre* mouse, which was mated again with mice carrying their respective homozygous floxed allele to produce either *Eif5a* ^*fl/fl*^; *Emx1-Cre* or *Dhps* ^*fl/fl*^;*Emx1-Cre* mice. *Camk2a-Cre* mediated KO of *Eif5a* or *Dhps* was achieved as above by the two-step breeding, using the *Camk2a-Cre* transgenic strain T29-1 (31). These four conditional KO (CKO) mice are referred to as *Eif5a*^*Emx*^, *Dhps*^*Emx*^, *Eif5a*^*Camk2a*^, and *Dhps*^*Camk2a*^, in the rest of the article. The genotypes of the CKO strains were confirmed by PCR as shown in Figure 2.

### The effects of temporal- and region-specific KO of *Eif5a* or *Dhps* in the brain on growth and survival of mice

**Figure 3:**
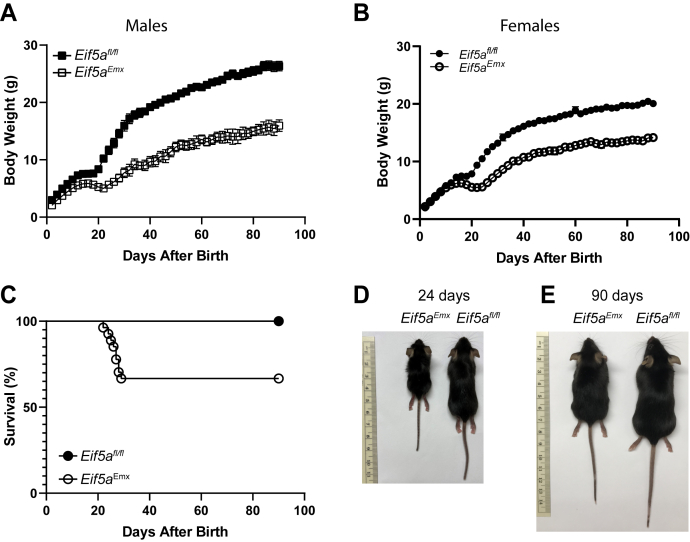
**Growth and viability of *Eif5a***^***Emx***^**and the control *Eif5a***^***fl/fl***^**mice.***A* and *B*, the body weights of the *Eif5a* CKO and the control mice were measured every 2 days for 90 days, and the male and female group body weights are plotted separately in panels *A* and *B*. The error bars represent the SEM. The numbers of mice were *Eif5a*^*fl/fl*^, M (n= 10) and F (n = 10); *Eif5a*^*Emx*^, M (n = 10), F (n = 17). *C*, viability of the *Eif5a*^*Emx*^ and *Eif5a*^*fl/fl*^ mice (male and female mice combined) in the 90 days after birth. The numbers of mice were *Eif5a*^*fl/fl*^ (n= 20) and *Eif5a*^*Emx*^ (n = 27). *D* and *E*, representative pairs of the *Eif5a* CKO and the control at 24 and 90 days after birth. The ruler is in centimeters. The body weights of the *Eif5a*^*Emx*^ and the control mice were 4.03 g (male) and 10.99 g (male) on day 24 and 13.32 g (female) and 21.24 g (female) on day 90. CKO, conditional KO.

In the *Emx1-Cre*–driven KO strains, the *Eif5a* or *Dhps* gene is downregulated in the neurons of the developing rostral brain including the cerebral cortex, and hippocampus, beginning at E9.5 and continuing throughout postnatal life. On the other hand, in the *Camk2a-Cre*–driven CKO strains, the expression of the target gene is abolished postnatally (beginning at P15–P21 and continuing through adulthood) in the *Camk2a*–expressing neurons in the CA1 regions of the hippocampus (31). Differential phenotypes were observed in all four CKO strains. Both male and female groups of *Eif5a*^*Emx*^ pups grew significantly slower than the control *Eif5a*^*fl/fl*^ pups (Fig. 3, *A* and *B*). There was little difference in the growth rates between the male and female groups of the *Eif5a*^*Emx*^ mice, whereas in the control *Eif5a*^*fl/fl*^ group, the males were consistently heavier than the female counterparts (Fig. 3, *A* and *B*). Moreover, survival was reduced in *Eif5a*^*Emx*^ mice compared with the control mice (Fig. 3*C*). The average body weights of both the male and female *Eif5a*^*Emx*^ mice were reduced compared with those of the control *Eif5a*^*fl/fl*^ mice throughout the period examined (Fig. 3, *A*–*D*)

**Figure 4:**
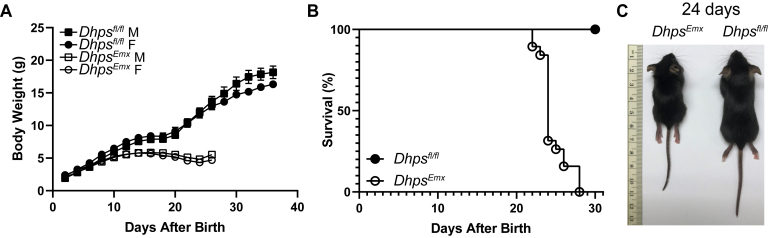
**Growth and viability of *Dhps***^***Emxl***^**and the control *Dhps***^***fl/fl***^**mice.***A*, the body weights of *Dhps*^*Emxl*^ and *Dhps*^*fl/fl*^ control, male, and female mice were measured every 2 days starting on day 2 after birth for 36 days until death. The numbers of mice in each group were *Dhps*^*fl/fl*^, M (n= 10) and F (n = 10) and *Dhps*^*Emxl*^, M (n = 11) and F (n = 8). The error bars represent the SEM. *B*, percent survival of *Dhps*^*Emxl*^ and control mice. The numbers of mice in each group were *Dhps*^*fl/fl*^ (n= 20) and *Dhps*^*Emxl*^ (n = 19). The n includes both male and female mice. *C*, representative pair of a *Dhps*^*Emxl*^ and a control *Dhps*^*fl/fl*^ mouse on day 24 after birth, with body weights of 5.92 g (male) and 10.62 g (female), respectively. The ruler is in centimeters.

At birth, the *Dhps*^*Emx*^ mice appeared to be similar in size to the control mice (*Dhps*^*fl/+*^, *Emx1-cre*, *Dhps*^*fl/fl*^, *Dhps*^*fl/+*^). However, the postnatal growth of *Dhps*^*Emx*^ mice was significantly impaired (Fig. 4*A*) and nearly arrested by day 12, whereas the control mice continued to grow. All *Dhps*^*Emx*^ pups died before 4 weeks after birth (Fig. 4*B*). On day 24, *Dhps*^*Emx*^ mice were much smaller than *Dhps*^*fl/fl*^ mice with the average whole-body weight less than 50% of the control mice (Fig. 4*C*). Unlike the deletion of *Eif5a* or *Dhps* in the Emx1-expressing neurons, deletion of either gene in the *Camk2a*-expressing neurons did not result in significant inhibition of growth, and no visible signs of developmental defects were observed in the first 3 months. However, both *Eif5a*^*Camk2a*^ and *Dhps*^*Camk2a*^ mice lost viability between 2 and 9 months of age (Fig. S1).

### The effects of deletion of *Dhps* or *Eif5a* on brain development and morphology

**Figure 5:**
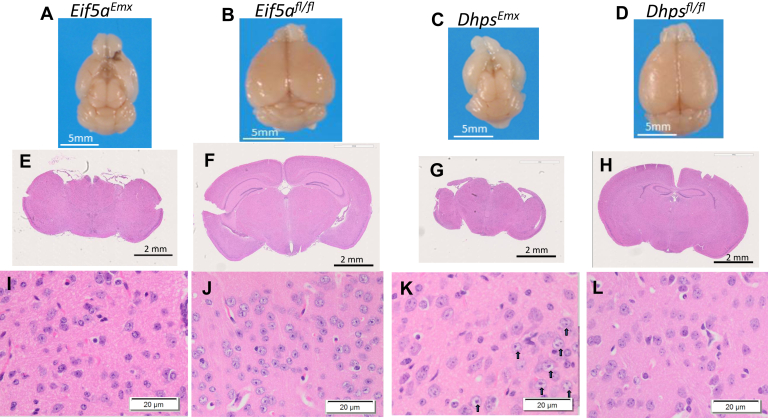
**Macroscopic and microscopic changes in the brains of *Eif5a***^***Emx***^**and *Dhps***^***Emxl***^**mice compared with their controls.***A*–*D*, a representative whole-brain image is shown for each strain: Both *Eif5a*^*Emx*^ and *Dhps*^*Emxl*^ brains show gross changes in the brain size and structures. *E*–*H*, a representative coronal section from each brain. *I*–*L*, microscopic images of the cortex region of coronal sections show cellular changes including vesiculated nuclei in *Dhps*^*Emxl*^ brains, as indicated by *black arrows*.

**Figure 6:**
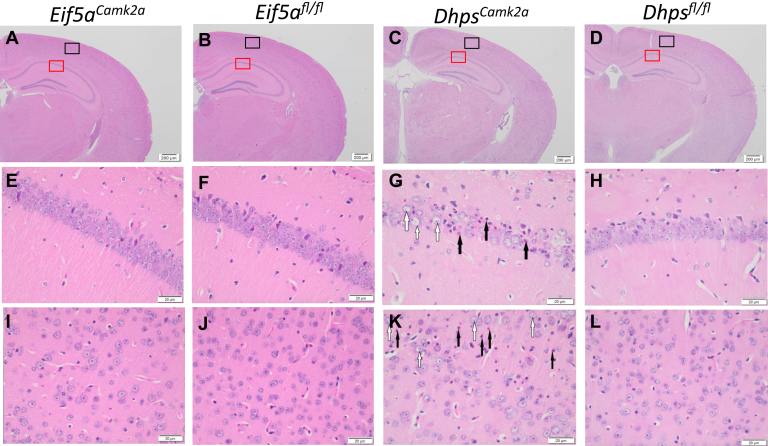
**Microscopic images of brain coronal sections of *Eif5a***^***Camk2a***^**and *Dhps***^***Camk2a***^**mice compared with their controls.***A*–*D*, part of coronal sections of *Eif5a*^*Camk2a*^, *Eif5a*^*fl/fl*^, *Dhps*^*Camk2a*^, *Dhps*^*fl/fl*^ mice. *E*–*H*, microscopic images of the CA1 region of the hippocampus in the *red box area*. *I*–*L*, microscopic images of the external cerebrum in the *black box area*. Necrotic neurons and vesiculated nuclei are indicated by *black* and *white arrows*, respectively.

The deletion of *Eif5a* or *Dhps* also exerted variable impacts on brain development in the four CKO strains (Figs. 5 and 6). Gross brain images revealed quite similar morphological defects in the brains of *Eif5a*^*Emx*^ and *Dhps*^*Emx*^ mice (Fig. 5, *A* and *C*), although *Dhps*^*Emx*^ mice displayed more serious defects in growth and survival than *Eif5a*^*Emx*^ mice. The average brain weight of the *Eif5a*^*Emx*^ mice at 4 months was less than that of controls (0.25 g *versus* 0.48 g, respectively). The same gross lesions shown in Figure 5*A* were observed in all *Eif5a*^*Emx*^ brains examined (1- and 4-month-old mice). The abnormal brain morphology included the loss of the cerebral cortex, hippocampus, corpus callosum, internal capsule, and portions of the lateral ventricles, and the opening of the third ventricle to the meninges. However, we could not detect cellular changes in the microscopic images of the remaining part of the *Eif5a*^*Emx*^ brain at 4 months (Fig. 5*I versus* Fig. 5*J*). The average weight of the *Dhps*^*Emx*^ brains was less than half of the control brain (0.19 g *versus* 0.431 g) on day 24. Each of the four *Dhps*^*Emx*^ brains examined showed the same gross abnormality (Fig. 5*C*), similar to that of the *Eif5a*^*Emx*^ brain (Fig. 5*A*). In the *Dhps*^*Emx*^ brain, the rostral portion of the cerebral cortex was missing or thinned. The deformity also included agenesis of the corpus callosum, hippocampus, internal capsule, and the distal portion of the cerebrum overlying the mid-brain. The lumen and the roof of the third ventricle were missing. Microscopic images of the remaining *Dhps*^*Emx*^ brain cerebrum showed the neurons enlarged and vesiculated (black arrows, Fig. 5*K*), not found in the control brain (Fig. 5*L*).

**Figure 7:**
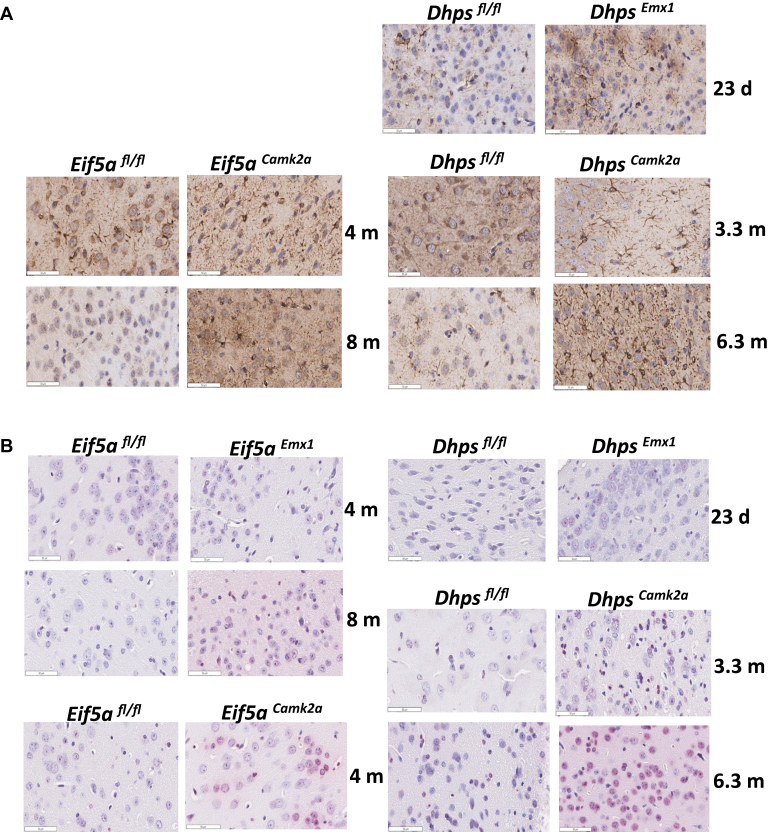
**Analysis of GFAP expression and TUNEL in brain tissue sections from *Eif5a* or *Dhps* CKO mice.***A*, the GFAP IHC was performed using the anti-GFAP primary antibody (Abcam, ab7260, 1:1000 dilution) as described under Experimental procedures with the brain slides of *Eif5a*^*Camk2a*^, *Dhps*^*Emx1*^, and *Dhps*^*Camk2a*^ mice and their respective controls, at different time points after birth. *B*, TUNEL assays were performed as described in Experimental procedures with the brain slides of *Eif5a*^*Emx1*^, *Dhps*^*Emx1*^, *Eif5a*^*Camk2a*^, and *Dhps*^*Camk2a*^ mice and their respective controls, at different time points after birth. CKO, conditional KO; GFAP, glial fibrillary acidic protein; IHC, immunohistochemistry; TUNEL, terminal deoxynucleotidyl transferase dUTP nick end labeling.

In contrast to the *Eif5a*^*Emx*^ and *Dhps*^*Emx*^ mice, *Eif5a*^*Camk2a*^ and *Dhps*^*Camk2a*^ mice appeared to grow normally and their gross brain images were indistinguishable from those of control mice (Fig. S2). However, microscopic examination revealed that *Dhps*^*Camk2a*^ mice had neuronal necrosis of the cerebral cortex and hippocampus at 4 months (Fig. 6, *G* and *K*). These regions contained necrotic neurons (black arrows) and neurons with enlarged nuclei with extensively vesiculated chromatin (white arrows, Fig. 6, *G* and *K*). Similar cellular changes were not observed in the *Eif5a*^*Camk2a*^ brains (Fig. 6, *E* and *I*). Interestingly, the immunostaining of glial fibrillary acidic protein, a marker of astrocyte, known to increase at the site of neuronal damage, appeared increased in the brain tissue sections from the *Eif5a*^*Camk2a*^, *Dhps*^*Emx*^, and *Dhps*^*Camk2a*^ mice at multiple postnatal ages (Fig. 7*A*). Furthermore, terminal deoxynucleotidyl transferase dUTP nick end labeling assays performed on brain tissue sections from the mutant mice and controls also showed the appearance of apoptotic cells (Fig. 7*B*).

### Impaired cognitive functions in the *Eif5a*^*Camk2a*^ and *Dhps*^*Camk2a*^ mice

**Figure 8:**
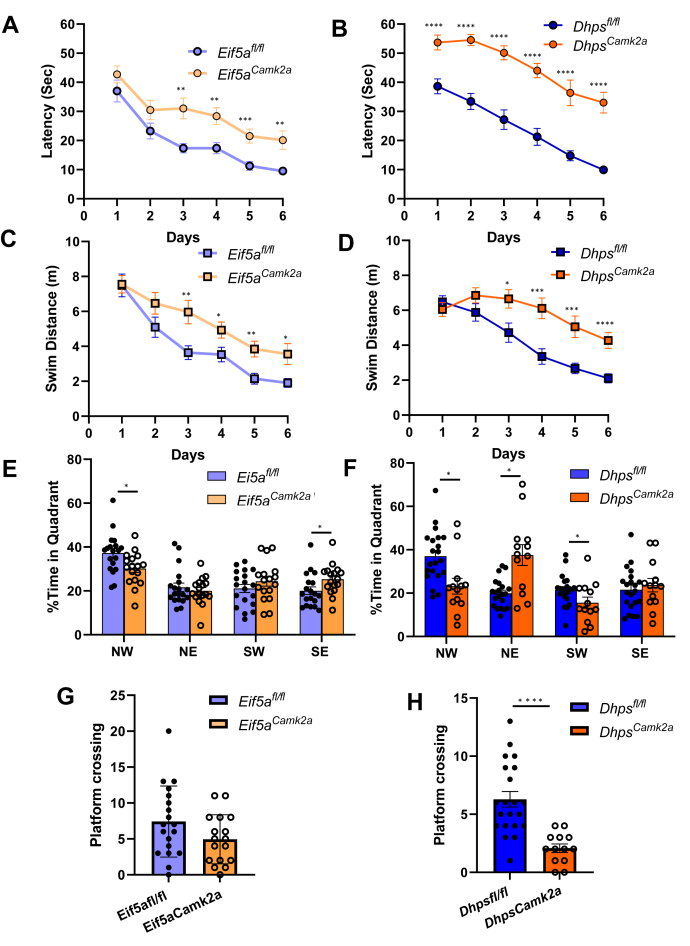
**Impaired spatial learning and memory in the *Eif5a***^***Camk2a***^**and *Dhps***^***Camk2a***^**mice.** The Morris water maze test was performed with the *Eif5a*^*Camk2a*^ and *Eif5a*^*fl/fl*^ control (*A*, *C*, *E*, and *G*) and *Dhps*^*Camk2a*^ and *Dhps*^*fl/fl*^ control (*B*, *D*, *F*, and *H*) as described under Experimental procedures. *A* and *B*, the average latency to the platform was significantly longer for the *Eif5a*^*Camk2a*^ (*A*) and *Dhps*^*Camk2a*^ (*B*) mice than their respective controls. *C* and *D*, the average swim distance to the platform by *Eif5a*^*Camk2a*^ (*C*) and *Dhps*^*Camk2a*^ (*D*) mice compared with their controls. *E* and *F*, the probe trial measured percentage time occupancy of *Eif5a*^*Camk2a*^ and *Eif5a*^*fl/fl*^ (*E*) and *Dhps*^*Camk2a*^ and *Dhps*^*fl/fl*^ (*F*) mice in the four quadrants. *G* and *H*, the probe trial also measured the number of crossings into the platform area by *Eif5a*^*Camk2a*^ and *Eif5a*^*fl/fl*^ (*G*) and *Dhps*^*Camk2a*^ and *Dhps*^*fl/fl*^ (*H*) mice. For panels *E*–*H*, the values are indicated as *closed circles* (controls) and *open circles* (CKO) and each bar represents the mean ± SD. The numbers of mice in each group were *Eif5a*^*Camk2a*^ (n = 17), *Eif5a*^*fl/fl*^ (n = 19), *Dhps*^*Camk2a*^ (n= 13), and *Dhps*^*fl/fl*^ (n = 21). Error bars represent the SEM. ∗*p* < 0.05, ∗∗*p* < 0.01, ∗∗∗*p* < 0.001, and ∗∗∗∗*p* < 0.0001 *via* Student’s *t* test. CKO, conditional KO.

We first compared the mobility of the *Eif5a*^*Camk2a*^ mice with that of the control mice by the open field test. The distance traveled in the whole arena, the mobile time, and the speed of the CKO mice were not reduced compared with the controls (Fig. S3), suggesting that their mobility was not impaired. Furthermore, all the *Eif5a*^*Camk2a*^ and *Dhps*^*Camk2a*^ mice displayed normal swimming ability 1 day before the Morris water maze (MWM) test. In the MWM test (Fig. 8), the mouse relies on visual cues to navigate to a submerged escape platform. Spatial learning was assessed by daily repeated trials for 6 days. The latencies to reach the hidden platform for the two controls, *Eif5a*^*fl/fl*^ and *Dhps*^*fl/fl*^, were 37.02 and 38.61 s, respectively, on day 1 and were shortened to 9.52 and 9.93 s, respectively, by day 6 (Fig. 8, *A* and *B*). On the other hand, the latencies of *Eif5a*^*Camk2a*^ and *Dhps*^*Camk2a*^ mice on day 1 (42.71 and 53.66 s, respectively) were longer than those of the respective controls, suggesting a poor baseline performance. Furthermore, the improvements of *Eif5a*^*Camk2a*^ and *Dhps*^*Camk2a*^ mice from day 1 to day 6 (reduction of latency by 53% and 38.5%, respectively) were much less than those of the respective control mice (reduction of latency by 74.3% and 75.1%, respectively), suggesting impaired learning in both CKO mice, with *DHPS*^*Camk2a*^ showing greater impairment than *EIF5A*^*Camk2a*^. The analyses of the swim distance to the hidden platform also provided a similar indication of learning disability of the two CKO strains (Fig. 8, *C* and *D*). The swim distances were similar for all four groups on day 1 but were significantly longer for the *Eif5a*^*Camk2a*^ and *Dhps*^*Camk2a*^ mice than their respective controls on consecutive days. The improvement indicated by a shortening of the swim distance from day 1 to day 6 was worse for the CKO groups than their controls, and *Dhps*^*Camk2a*^ consistently underperformed *Eif5a*^*Camk2a*^ mice. These results provide strong evidence that both *Eif5a*^*Camk2a*^ and *Dhps*^*Camk2a*^ mice are impaired in spatial learning and that the impairment is more severe in *Dhps*^*Camk2a*^ than in *Eif5a*^*Camk2a*^ mice (Fig. 8, *A*–*D*).

After the completion of the 6-day trials, reference memory was evaluated by a probe trial after the removal of the hidden platform. The percentage time occupancy in the target quadrant (North West [NW]) (Fig. 8, *E* and *F*) and the number of crossings into the small area that previously contained the removed platform were measured (Fig. 8, *G* and *H*). The *Eif5a*^*fl/fl*^ and *Dhps*^*fl/fl*^ control groups occupied the target NW quadrant for a significantly longer time, (37.04% and 37.21% time in quadrant, respectively) than in three other quadrants (∼20% in each quadrant). In contrast, the preference to occupy the target quadrant was significantly reduced in *Eif5a*^*Camk2a*^ mice compared with the controls (30.18% *versus* 37.21%) and in *Dhps*^*Camk2a*^ mice compared with the controls (23.20% *versus* 37.04%), suggesting the impaired memory in both CKO groups. Curiously, *Dhps*^*Camk2a*^ mice tended to occupy the South West instead of NW quadrant (Fig. 8*F*). On the probe trial, the mice were placed in the pool at the boundary of the North East and South East quadrants, or east start point. The control mice quickly and more directly moved to the NW quadrant to search for the platform location, suggesting good memory of the location while the mutant mice spent more time searching in the North East and South East quadrants before moving to search in the NW location, as shown in the track plots (Fig. S4), consistent with poor memory of the platform location during the probe trial. The average numbers of crossings into platform area were lower in the CKO mice than in the controls (7.42 and 4.94, respectively, for *Eif5a*^*fl/fl*^ and *Eif5a*^*Camk2a*^ mice and 6.29 and 2.08, respectively, for *Dhps*^*fl/fl*^ and *Dhps*^*Camk2a*^ mice). Both the platform occupancy and the platform area entry data provide clear evidence for memory impairment in the CKO mice, *Dhps*^*Camk2a*^ mice being more deficient than *Eif5a*^*Camk2a*^ mice.

**Figure 9:**
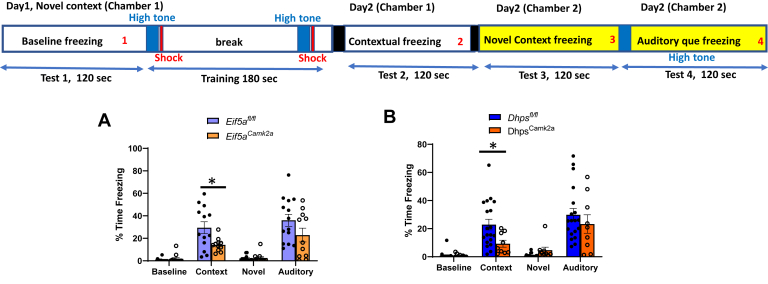
**Impaired contextual learning in the *Eif5a***^***Camk2a***^**and *Dhps***^***Camk2a***^**mice.** (*A*, *B*), The contextual learning test was performed with the two CKO mice and their control mice, as described in Experimental procedures. Baseline freezing, contextual freezing, novel context freezing, and auditory cue freezing were measured. The values are indicated as *closed circles* (controls) and *open circles* (CKO), and each bar represents the mean ± SD. The numbers of mice in each group were *Eif5a*^*Camk2a*^ (n = 11), *Eif5a*^*fl/fl*^ (n = 14), *Dhps*^*Camk2a*^ (n = 9), and *Dhps*^*fl/fl*^ (n = 19). The error bars represent the SEM. ∗*p* < 0.05, ∗∗*p* < 0.01, and ∗∗∗*p* < 0.001 *via* Student’s *t* test. CKO, conditional KO.

Then, a contextual learning (cued fear conditioning) test was carried out as outlined in the top panel of Figure 9. The baseline freezing and novel context baseline freezing were low and no significant differences were observed among the four groups of mice. However, contextual freezing time was significantly reduced in *Eif5a*^*Camk2a*^ mice (to 48% of the control *Eif5a*^*fl/fl*^ value) and *Dhps*^*Camk2a*^ (to 40% of *Dhps*^*fl/fl*^ value) (Fig. 9, *A* and *B*). Auditory cued freezing was also reduced in *Eif5a*^*Camk2a*^ mice (to 64% of the control) and in *Dhps*^*Camk2a*^ mice (to 78% of the control), but not as much as the contextual freezing. Taken together, the data in Figures 8 and 9 clearly demonstrate the impairment in spatial learning, memory, and contextual learning in mice in which *Eif5a* or *Dhps* is deleted in the *Camk2a*-expressing neurons of the cortex and hippocampus.

## Discussion

Recent genetic studies have provided evidence that certain variants in the *EIF5A* (27) or *DHPS* gene (28) are associated with rare neurodevelopmental disorders in humans. Furthermore, individuals with *DOHH* variants who display similar developmental delay and intellectual disability have also been identified (Ziegler A. *et al.*, unpublished results), underscoring the importance of each step of the hypusine modification pathway and thereby the critical role of the hypusinated eIF5A in neurodevelopment in humans (29). These findings led us to generate the four CKO mouse models, with temporal- and region-specific deletion of either *Eif5a* or *Dhps* in the forebrain, and to assess the impact on development. Different phenotypes in brain development, growth, survival, and cognitive functions were observed in these CKO strains depending on the targeted gene and the Cre driver. Although the CKO strains do not harbor the same variants of the *EIF5A* and the *DHPS* genes as those of the affected human individuals, it is interesting to note that certain features of the human neurodevelopmental disorders, including intellectual disability, developmental delay, reduced growth, and shortened lifespan, are reflected in the phenotypes of these CKO mice.

The deletion of *Eif5a* or *Dhps* in the *Emx1*-expressing neurons from the mid-embryonic stage resulted in gross morphological abnormalities in the brain; the cerebral cortex was thinned or missing and the hippocampus, corpus callosum, and the internal capsule portions of the ventricles were also missing because of agenesis (Fig. 5, *A* and *C*). These results indicate that both *Eif5a* and *Dhps* are essential for the embryonic and postnatal development of the cortex and hippocampus. Although gross brain defects were similar in the *Eif5a*^*Emx*^ and the *Dhps*^*Emx*^ mice, the deleterious effects on growth and viability were more severe in *Dhps*^*Emx*^ mice than in *Eif5a*^*Emx*^ mice. Approximately 67% of *Eif5a*^*Emx*^ mice survived longer than 3 months, whereas all *Dhps*^*Emx*^ mice died within 4 weeks after birth. The behavioral tests were not performed on these groups of CKO mice because of their short life spans and premature and unpredictable death, especially of *Dhps*^*Emx*^ mice.

Whole-body KO of *Eif5a* or *Dhps* causes an early embryonic lethality (between E3.5 and E6.5) (6). It is predicted that eIF5A and DHPS would be depleted in the cortex and hippocampus area within a few days after Emx1-Cre expression at E.9.5. Given this timeline for gene deletion and the resultant observed phenotype, the mechanisms underlying the loss of the cortex and hippocampus may involve the failure of neural stem cells to proliferate or to differentiate, apoptosis of differentiating cells, or a failure of differentiating cells to proliferate. Previous studies using mouse and zebrafish models of DHPS knockdown during the developmental and postnatal periods have demonstrated that loss of DHPS impacts mRNA translation, which in turn disrupts cellular differentiation, organ development, and/or cellular proliferation (7, 8, 32). Therefore, as a translation elongation factor affecting synthesis of a wide array of cellular proteins, depletion of hypusinated eIF5A may affect multiple cellular processes in brain development.

The postnatal ablation of *Eif5a* or *Dhps* in the *Camk2a*-expressing neurons did not cause gross changes in the brain compared with those found in the *Eif5a*^*Emx*^ and the *Dhps*^*Emx*^ mice, suggesting that the development of the cortex and hippocampus was unaltered. Although no significant growth inhibition or visible defects were found in *Eif5a*^*Camk2a*^ or *Dhps*^*Camk2a*^ mice, they both died prematurely between 2 and 9 months. Furthermore, they displayed concrete evidence of impairment in cognitive functions (Figs. 8 and 9), *Dhps*^*Camk2a*^ being more affected than *Eif5a*^*Camk2a*^ mice. These cognitively impaired CKO mice hold potential utility in the future development of chemical or biological therapeutics for human neurodevelopmental disorders caused by variants of *EIF5A*, or *DHPS*.

Common phenotypes between *Eif5a*^*Emx*^ and *Dhps*^*Emx*^ mice and between *Eif5a*^*Camk2a*^ and *Dhps*^*Camk2a*^ mice strongly suggest that a common mechanism underlies the impairment in both CKO mice. However, it is hard to explain why the ablation of *Dhps* is more detrimental than that of *Eif5a*, as is evident in all the observed phenotypes. This is counterintuitive, as the hypusinated eIF5A is the direct player in translation elongation, whereas DHPS is a modifier of eIF5A activity. One possibility may be that the eIF5A2 isoform (modified to the hypusine form) can partially compensate for the loss of eIF5A in the targeted neurons of the mouse brain. However, we did not find clear evidence for accumulation of the eIF5A2 isoform protein in brain tissues of control or CKO mice (data not shown). The whole-body KO of eIF5A is embryonic lethal in mouse, suggesting that eIF5A2 is not induced during early embryonic development upon KO of eIF5A and that eIF5A2, in this scenario, may not compensate for the loss of eIF5A (6). It is also possible that there are differences in the efficiency of the Cre-mediated recombination at the two different target gene loci. Whereas knockdown of expression of eIF5A or DHPS has been carefully validated for both conditional mouse alleles using other cell-specific cre-mediated models (7, 8) and the efficiency of the Emx1-cre and Camk2a-cre drivers has been published (30, 31), our study did not perform a direct comparison of the recombination efficiencies of these alleles in our models. Another possibility is the interference of activities of hypusinated eIF5A by unhypusinated eIF5A precursors that accumulate upon depletion of spermidine or upon inhibition of DHPS. Although the unhypusinated eIF5A precursors were inactive and did not appear to interfere with the activity of hypusinated eIF5A in the *in vitro* assays of methionyl-puromycin synthesis (33), their potential effects on translation need to be reevaluated *in vivo*. It is possible that the eIF5A precursors still associate with the 80S ribosome through interactions not involving the hypusine residue (19) and interfere with the action of hypusinated eIF5A in mammalian cells and tissues. In such a case, the potential interference by the eIF5A precursors that may have accumulated in *Dhps*^*Emx*^ and *Dhps*^*Camk2a*^ brains could explain their more deleterious phenotypes. In the case of human patients, a heterozygous *de novo EIF5A* variant with partial activities causes clinical phenotypes (27), suggesting that proper neuronal function in humans cannot tolerate even a partial loss (<50%) of eIF5A activity. The detrimental effects of heterozygous *EIF5A* variants may not be simply due to a reduction in active eIF5A but may also be compounded by the interference by the eIF5A variants. The molecular basis underlying the better survival and performance of the *Eif5a* CKO mice than the *Dhps* CKO mice warrants further investigation.

The implication of variants of *EIF5A* or *DHPS* in human neurodevelopmental disorders is not surprising in view of the fact that variants in a number of other factors in the translation machinery such as alanyl tRNA synthetase and eukaryotic translation elongation factors 2 (EF2) and 1a2 (EF1a2) have been associated with neurodevelopmental disorders (34). Errors during translation elongation can lead to production and accumulation of aberrant proteins that are toxic to neural cells. In human individuals with variants in *EIF5A*, or *DHPS*, major clinical symptoms were associated with neurodevelopment (27, 28), suggesting that neuronal systems are most vulnerable to a deficiency in hypusinated eIF5A. Global proteomics analyses provided evidence that depletion of eIF5A in mammalian cells led to endoplasmic reticulum stress, unfolded protein response, and upregulation of chaperone expression (35). In addition to these general effects of eIF5A depletion, it is also possible that there are key regulatory factors of brain development that may be specifically dependent on eIF5A for translation. Future studies will be directed toward elucidation of molecular mechanisms underlying these neurodevelopmental disorders stemming from a reduction in bioactive, hypusinated eIF5A and the identification of downstream effectors of eIF5A.

## Experimental procedures

### Mouse maintenance and sample collection

All experimental procedures involving mice were approved by the National Institute of Dental and Craniofacial Research Animal Care Committee and were conducted in accordance with approved protocols. Pups were housed in an animal facility with a 14/10 h light/dark cycle in positive pressure–ventilated racks with filtered-top cages (Lab Products Inc). Animals were fed autoclavable rodent pellets, NIH-07 Mouse/Rat Diet (Envigo, #7022) and UV-treated ultra-filtered water *ad libitum* throughout the experiments. To help the feeding of small mutant mice, hydrogel and soft foods were offered in petri dishes on the floor of the cages.

### Mouse strains used for brain-specific KO of *Eif5a* or *Dhps*

The conditional mouse strains used, *Eif5a*^*fl/fl*^ and *Dhps*^*fl/fl*^ (Dhpstm1.1Mirm/J, stock #034895, Jackson Laboratory), were previously reported (7, 8). Neither the *Eif5a*^*fl/fl*^ mice nor *Dhps*^*fl/fl*^ mice showed phenotypic differences compared with their WT C57BL/6 littermates, and as a result, *Eif5a*^*fl/fl*^ and *Dhps*^*fl/fl*^ mice were used as controls for the respective CKO strains. The homozygous *Emx1-IRES-Cre* (B6.129S2-Emx1, Jackson Laboratory) (30) were viable, fertile, and normal in size and did not display any gross physical or behavioral abnormalities. Recombination occurs in approximately 88% of the neurons of the neocortex and hippocampus and in the glial cells of the pallium starting at E.9.5. The Camk2a transgenic strain, T29-1 (B6.Cg-Tg(Camk2a-Cre), Jackson Laboratory) used in the study displayed a normal phenotype and the Camk2a-Cre recombinase was expressed in the forebrain, predominantly in the CA1 pyramidal cell layer in the hippocampus postnatally 3 to 4 weeks after birth (31).

### Genotyping of KO mice

Genomic DNA was isolated from tail biopsies using the QIAamp DNA Blood Mini Kit (QIAGEN) according to the manufacturer’s instruction. For PCR analysis, mouse tail DNA was amplified using JumpStart Taq ReadyMix (MilliporeSigma). The *Dhps* lox PCR and *Eif5a* lox PCR were carried out as described previously (7, 8).

### Histochemical analysis

The animals were euthanized with carbon dioxide. A necropsy was performed, and multiple tissues and organs were collected and placed in 10% formalin and fixed for 24 to 48 h. The tissues were then processed through a series of alcohols and xylenes and embedded in paraffin.

Serial sections (thickness of 10 μm) were prepared and stained with 0.1% H&E or used for immunohistochemistry at Histoserv Inc as follows: Slides were deparaffinized and hydrated through graded alcohols to distilled water, followed by antigen retrieval. They were then blocked with hydrogen peroxide and a blocking serum, and slides were washed in distilled water. Next, the slides were incubated with the primary antibody, a secondary antibody, and horseradish peroxidase-conjugated streptavidin. Finally, the slides were developed using 3,3′-diaminobenzidine and counterstained with hematoxylin. All of the incubations were carried out at room temperature (RT) and TBST was used as a washing buffer.

### Terminal deoxynucleotidyl transferase dUTP nick end labeling assays

Formalin-fixed paraffin-embedded brain tissue sections mounted on glass slides were deparaffinized and hydrated through graded alcohols to distilled water, followed by proteinase digestion at 37 °C. Tissue sections were then blocked with a blocking serum, rinsed, and transferred to a buffer solution. Next, the slides were incubated in the TdT/dUTP reaction mixture at 37 °C. The tissue sections were again blocked with a blocking serum, rinsed, and detected with an anti-digoxigenin detection system. Finally, the slides were developed with Vector Red and counterstained with hematoxylin. Unless otherwise specified, incubations were performed at RT, and TBST was used as a washing buffer.

### Open field test

To measure the general activities of mice, the open field test was performed as follows: mice were removed from their home cages and gently placed in a 16” × 16” × 16” Perspex arena viewing chamber and their movement was recorded for periods ranging from 5 min to 30 min, and then, the mice were returned to their standard home cages. The recordings were fed into a software program that analyzes the mouse behavior and movement.

### MWM test

Learning plasticity and cognitive flexibility were tested by the MWM test (36) with minor modifications. Although the ages of tested mice varied from 2.5 to 5 months, a mutant and a matching control (with the same or close to the same age) were set up to be tested in pairs. The MWM test was carried out in a circular pool (4 ft. diameter, 30 in. high, San Diego Instruments) filled with water, which was made opaque with addition of non-toxic white paint (Crayola) and kept at 20 to 30 °C. A small, square, clear plexiglass escape platform (hidden platform) was placed in the NW quadrant of the tank, 1 cm beneath the water surface. The swim distance, latency to find the platform, swim speed, path length to platform, and so forth were measured using behavioral tracking software (ANY-maze). The mice received four learning trials per day (trials lasted maximum of 60 s) on six consecutive days. After learning trials were completed, a probe trial was performed on the last day, in which the platform was removed. The number of crossings over the location in the pool previously occupied by the removed platform and the percentage of time spent in each of the four pool quadrants were measured for 90 s.

### Contextual learning test

The test was conducted following guidelines of a published protocol (37) with modifications. While the animal was in the chamber and provided cues, the following data including the total freezing time, number of freezing episodes, duration of freezing episodes, and latency between stimuli and freezing were collected. Mice were placed in a sound-attenuating chamber, 17 cm × 17 cm × 25 cm (w, d, h) with a light and speaker. After baseline freezing was measured for 120 s, auditory tones (2 × 4 kHz 80 dB tone) were delivered into the chamber for 15 s, followed by a 2-s foot shock (0.85 mA) through the grid floor. After a break for 120 s, the tone-shock procedure was repeated, and the mice were returned to their cages. On day 2 (24th hour), the mice were re-exposed to the chamber used on day 1 and contextual freezing was recorded. Afterward (25th hour), the mice were placed in a completely new chamber and novel context freezing was measured for 120 s. Then, auditory cues were applied and auditory cue freezing was measured.

### Statistics

All data are presented as the mean ± SEM and were analyzed using the software GraphPad Prism 5.0 (GraphPad Software) and OriginPro (OriginLab Corp). Student’s *t* test was applied to determine the significance between two groups. ANOVA with the post hoc Tukey test was used to analyze the time in each chamber in the social behavior test and the time in quadrants in the MWM. Two-way ANOVA was applied to compare the probe trial data in the MWM and in the contextual learning test. Statistical significance was defined at *p* < 0.05 and presented as ∗*p* < 0.05, ∗∗*p* < 0.01, ∗∗∗*p* < 0.001, and ∗∗∗∗*p* < 0.0001.

## Data availability

All the data described are contained within the article.

## Supporting information

This article contains supporting information.

## Conflict of interest

The authors declare that they have no conflicts of interest with the contents of this article.

## Footnotes

^§^ In mammals, there are two eIF5A genes, encoding highly conserved isoforms, eIF5A1 and eIF5A2, and both undergo hypusine modification. As eIF5A1 is the isoform predominantly expressed, eIF5A commonly represents eIF5A1. eIF5A can also be used to represent both forms collectively.
