# Supplementary material for: Neuron-specific ablation of eIF5A or deoxyhypusine synthase leads to impairments in growth, viability, neurodevelopment, and cognitive functions in mice
Source: J Biol Chem. 2021 Oct 22;297(5):101333. doi: 10.1016/j.jbc.2021.101333 (PMC8605248; doi:10.1016/j.jbc.2021.101333)

Supporting Information

Neuron specific ablation of eIF5A or deoxyhypusine synthase leads to impairment in growth, viability, neurodevelopment and cognitive functions in mice

Rajesh Kumar Kar, Ashleigh S. Hanner, Matthew F. Starost, Danielle Springer, Teresa L. Mastracci, Raghavendra G. Mirmira, Myung Hee Park

Fig. S1

Fig. S2

Fig. S3

Fig. S4

Figure legends

Figure S1. Reduced survival of $Eif5a^{Camk2a}$ and $Dhps^{Camk2a}$ mice. *A, B*, The survival curves of $Eif5a^{Camk2a}$ (*A*) and $Dhps^{Camk2a}$ (*B*) mice show reduced viability of both CKO mice compared to the respective controls, $Eif5a^{fl/fl}$, and $Dhps^{fl/fl}$.

Figure S2. Macroscopic images of brains of $Eif5a^{Camk2a}$ and $Dhps^{Camk2a}$ mice in comparison with the respective controls. The brains were taken from $Eif5a^{Camk2a}$ and $Eif5a^{fl/fl}$ mice (both 8 months old) and from $Dhps^{Camk2a}$ and $Dhps^{fl/fl}$ mice (both 3 months old).

Figure S3. Open field test of $Eif5a^{Camk2a}$ and $Eif5a^{fl/fl}$. To measure the general mobility of mice, open field testing was performed as described under Experimental Procedures. Mice were placed in a 16" X 16" X 16" Perspex arena viewing chamber and their movement was recorded for 30 minutes. The travel distance (*A*), the mobile time (*B*) and the speed (*C*) of the mice in the arena are shown. The numbers of mice in each group were $Eif5a^{Camk2a}$ (n=10), $Eif5a^{fl/fl}$ (n=11). Error bars represent SEM. There was no significant difference between the control and $Eif5a^{Camk2a}$ groups in travel distance, mobile time, and speed.

Fig. S4. Morris Water Maze probe trial track plots. *A*, Representative track plots from $Dhp^{fl/fl}$ control mice show a focused search strategy with increased activity in the NW quadrant. *B*,

Representative track plots from *Dhps*^{Camk2a} mice show less crossings over platform location, less search activity in the NW quadrant, and increased activity east of the platform closer to the start location of the test.

Fig. S1.

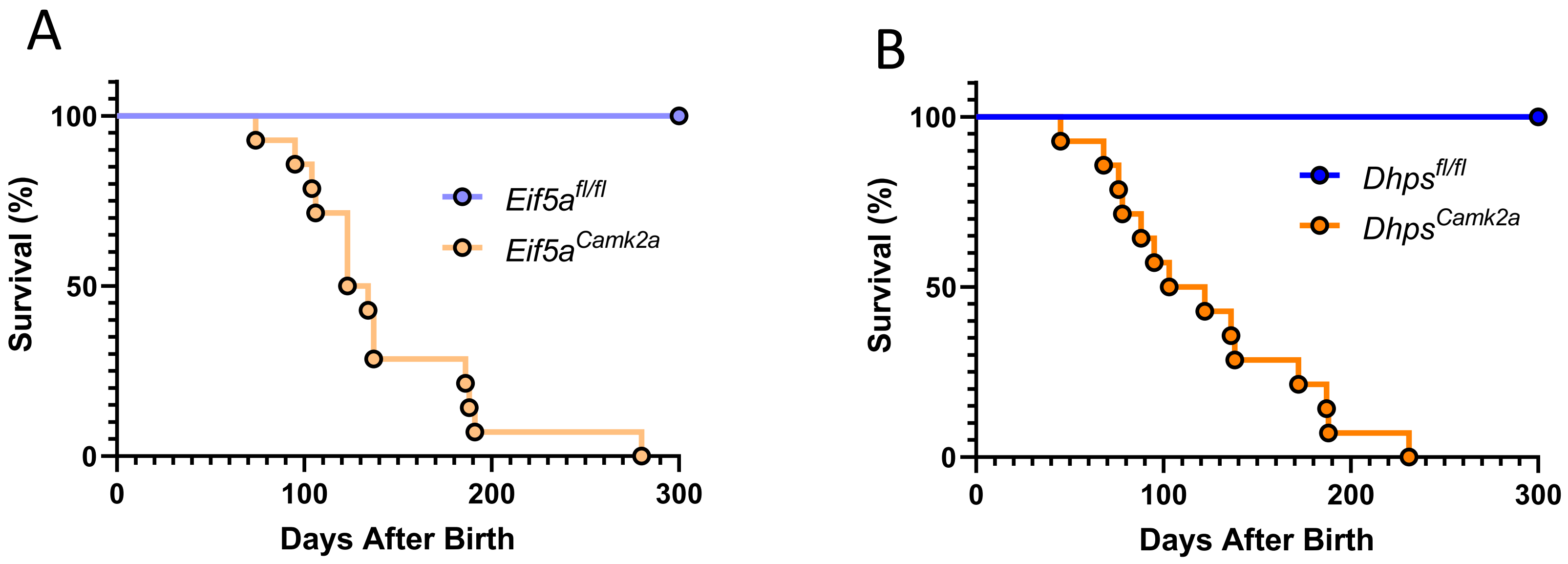
A
100
50
0
Survival (%)
0
100
200
300
Days After Birth
Eif5a fl/fl
Eif5a Camk2a
B
100
50
0
Survival (%)
0
100
200
300
Days After Birth
Dhps fl/fl
Dhps Camk2a

Fig. S2.

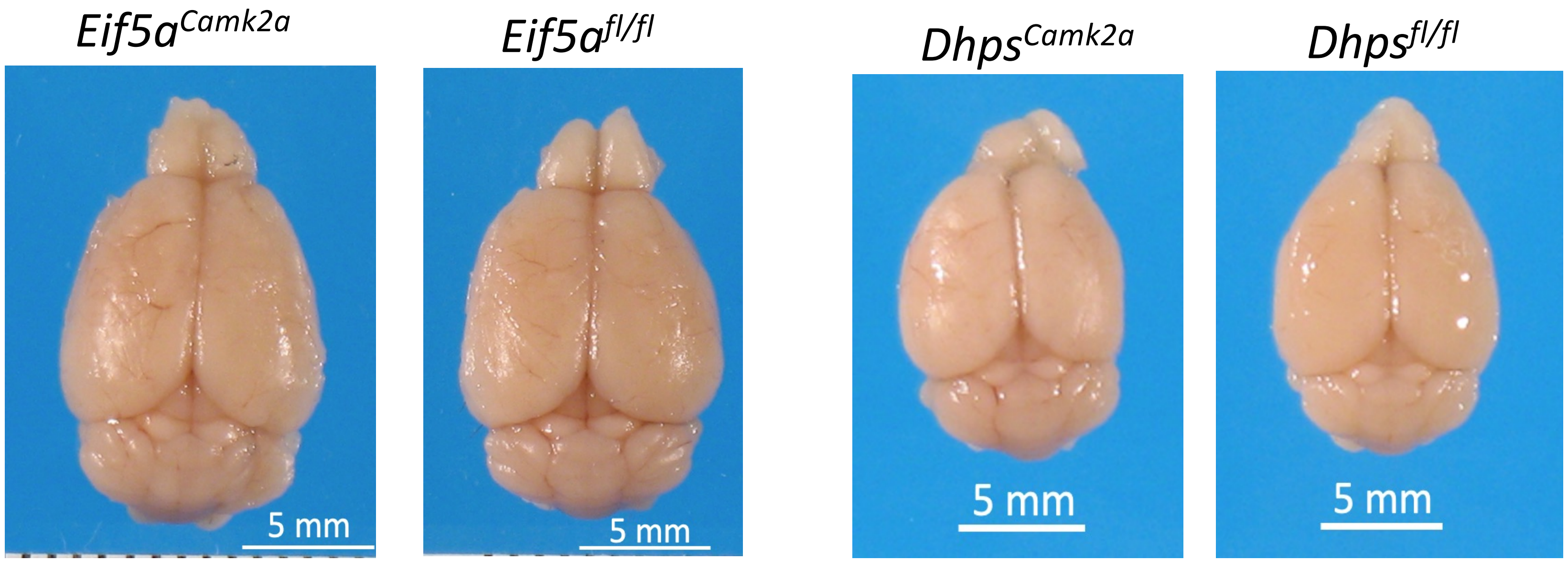
Eif5a^{Camk2a}
Eif5a$^{fl/fl}$
Dhps^{Camk2a}
Dhps$^{fl/fl}$
5 mm
5 mm
5 mm
5 mm

Fig. S3, Open field test

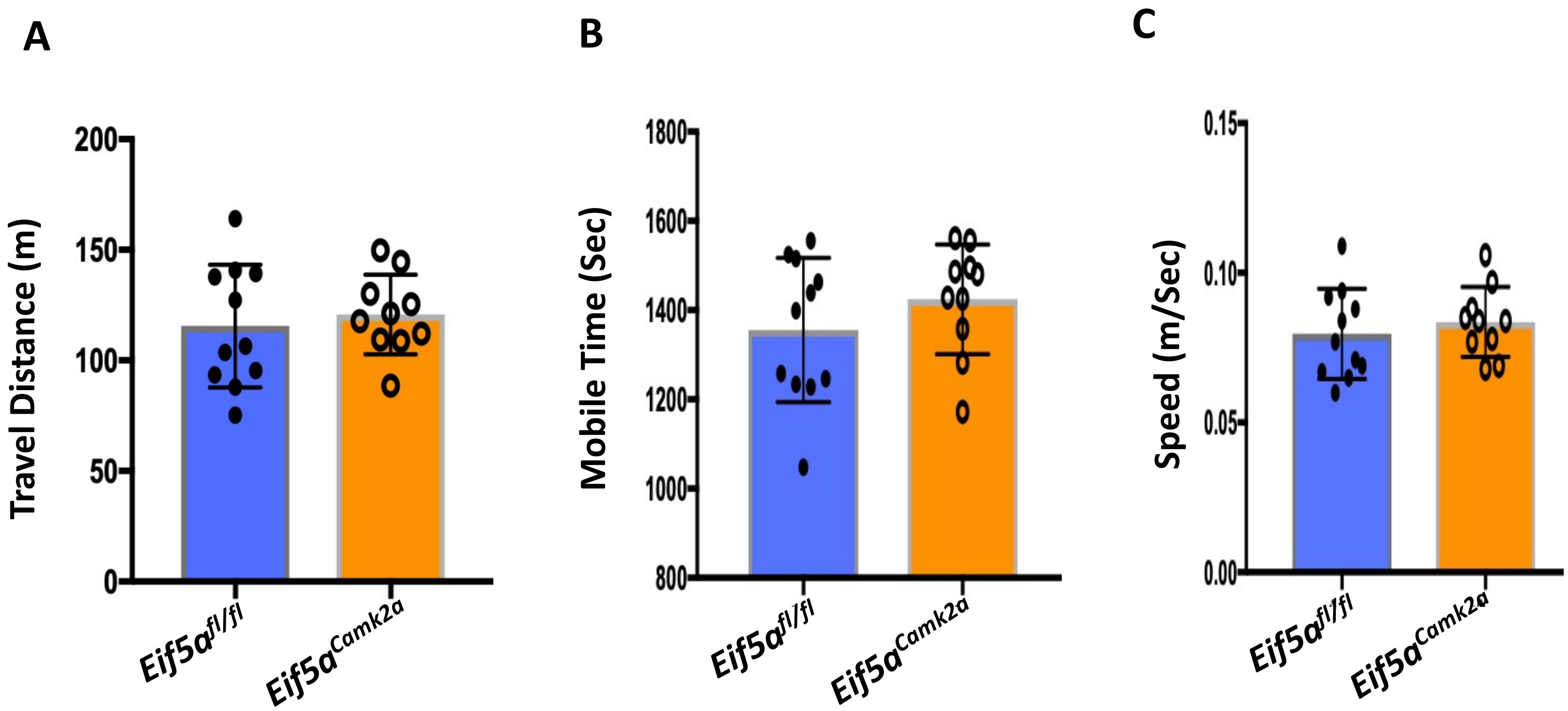

Fig. S4, MWM probe trial track plots

A *Dhps*$^{fl/fl}$

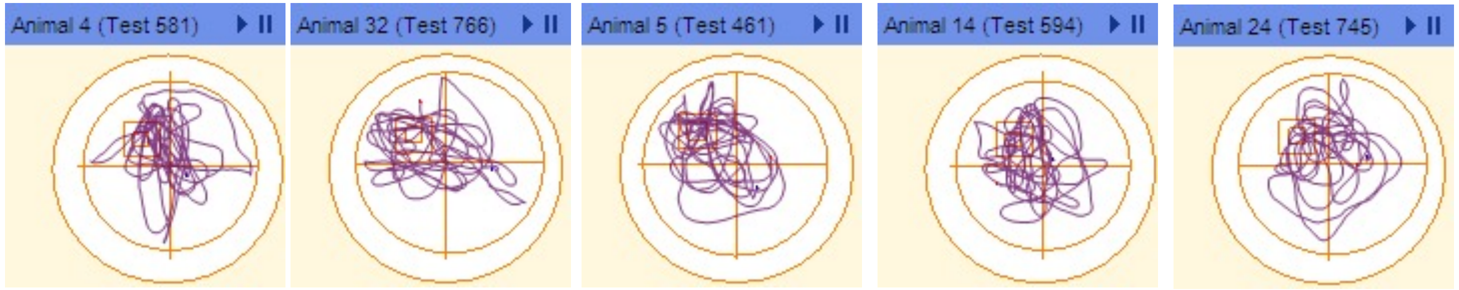

B *Dhps*^{Camk2a}

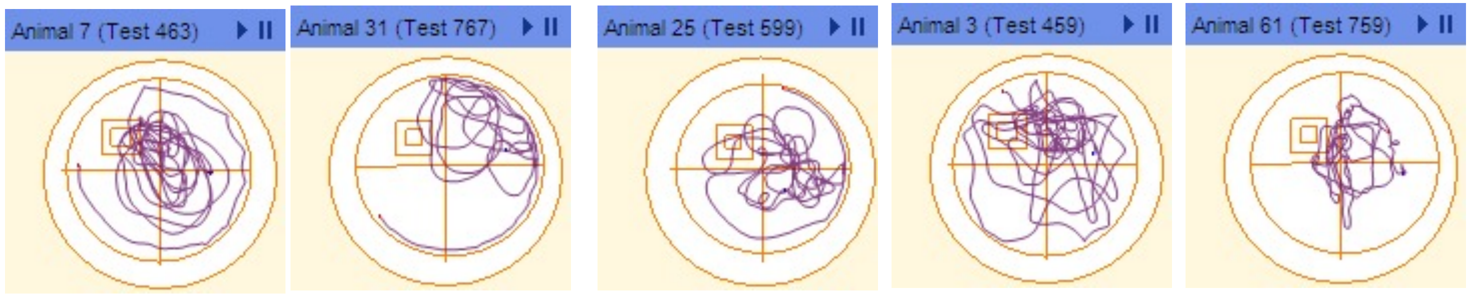

Supplement: Supplemental Figures S1–S4 [file mmc1.pdf]
